# Cesarean deliveries and maternal weight retention

**DOI:** 10.1186/s12884-017-1527-x

**Published:** 2017-10-04

**Authors:** Kandice A. Kapinos, Olga Yakusheva, Marianne Weiss

**Affiliations:** 1 RAND Corporation, 1200 South Hayes, Arlington, VA 22202 USA; 2 University of Michigan, School of Nursing and School of Public Health, 400 North Ingalls Building, Ann Arbor, MI 48109-5482 USA; 3 Marquette University College of Nursing, 530 N 16th St, Milwaukee, WI 53233 USA

**Keywords:** Cesarean delivery, Post pregnancy weight, Fetal malpresentation

## Abstract

**Background:**

Cesarean delivery accounts for nearly one-third of all births in the U.S. and contributes to an additional $38 billion in healthcare costs each year. Although Cesarean delivery has a long record of improving maternal and neonatal mortality and morbidity, increased utilization over time has yielded public health concerns and calls for reductions. Observational evidence suggests Cesarean delivery is associated with increased maternal postpartum weight, which may have significant implications for the obesity epidemic. Previous literature, however, typically does not address selection biases stemming from correlations of pre-pregnancy weight and reproductive health with Cesarean delivery.

**Methods:**

We used fetal malpresentation as a natural experiment as it predicts Cesarean delivery but is uncorrelated with pre-pregnancy weight or maternal health. We used hospital administrative data (including fields used in vital birth record) from the state of Wisconsin from 2006 to 2013 to create a sample of mothers with at least two births. Using propensity score methods, we compared maternal weight prior to the second pregnancy of mothers who delivered via Cesarean due to fetal malpresentation to mothers who deliver vaginally.

**Results:**

We found no evidence that Cesarean delivery in the first pregnancy causally leads to greater maternal weight, BMI, or movement to a higher BMI classification prior to the second pregnancy.

**Conclusions:**

After accounting for correlations between pre-pregnancy weight, gestational weight gain, and mode of delivery, there is no evidence of a causal link between Cesarean delivery and maternal weight retention.

## Background

Cesarean delivery is the most common surgery in the U.S. occurring in nearly 33% of all live births [1, 2]. Rates of Cesareans have increased from a low of 21% in 1996 to almost 33% where the rate has remained since 2009 [3, 4]. For nearly 3 decades, the World Health Organization (WHO) has argued that although Cesarean deliveries clearly improve maternal and child health outcomes when medically indicated, a population-level Cesarean section rate of greater than 10–15% is not linked with better health outcomes [5]. Evidence suggests Cesarean delivery when *not* medically indicated leads to negative health outcomes - longer postpartum recovery [6], higher rates of re-hospitalization [7], longer hospital stays [8] and greater risk of maternal morbidity [9]. Beyond the impact on the immediate pregnancy outcomes, there may be longer-term adverse impacts on maternal health outcomes, including cumulative weight retention between pregnancies [10, 11].

Several observational studies have documented evidence of an association between Cesarean delivery and subsequent maternal weight. The odds of Cesarean delivery are 1.46 and 2.05 higher for women who are overweight or obese prior to their first pregnancies, respectively, relative to normal weight women [11]. There is also weak-to-moderate evidence on the positive association between gestational weight gain and Cesarean delivery suggesting that greater gestational weight gain is linked to a higher risk of Cesarean delivery even controlling for comorbidities [7, 12–16]. For example, one study using US vital statistics records reported a nearly 40% increase in the adjusted odds of Cesarean delivery for women who gained more than 40 lb during pregnancy [13].

The challenge in identifying a causal effect of Cesarean delivery on maternal weight is that many of the medical indications for Cesarean delivery are also independently associated with both pregravid and postpartum weight. The most common clinical indications for Cesarean delivery are labor dystocia (slow labor) and non-reassuring fetal tracing (interderminate fetal heart), occurring in 34 and 23% of all Cesarean deliveries, respectively [17]. The likelihood of slower labor progression and fetal distress both increase as the mother’s pre-pregnancy body mass index (BMI) increases [15, 18, 19]. Pre-pregnancy weight or BMI is also correlated with both gestational weight gain and postpartum weight [15]. Therefore, we might observe a positive association between Cesarean delivery and maternal postpartum weight simply because heavier women are at a greater risk of having the clinical indications for Cesarean delivery and are more likely to weigh more after birth. Therefore, a positive association of Cesarean delivery with maternal postpartum weight retention may be driven by this confounding and not necessarily by the Cesarean itself.

Improved knowledge on the causal effect of Cesarean delivery may better inform provider practices and maternal choices, which can result in a healthier population and may generate significant healthcare cost savings. If Cesareans causally result in greater weight retention for new mothers, for example, due to slower physical recovery, then intervention efforts could focus on removing post-operative barriers. If, instead, the positive association between Caesareans and maternal post-birth weight is really driven by the fact that women who are heavier prior to pregnancy are more likely to have a Cesarean, then that adds to the argument that broader and more intensive efforts to promote healthier lifestyle and weight habits throughout the life course are needed (irrespective of pregnancy). Maternal post-birth weight retention is of significant concern for the growing obesity problem in the U.S. with studies documenting permanent weight retention of 13 to 22 lb for mothers 10 to 15 years after giving birth, [20, 21] the magnitude of which can easily move a woman from normal weight to overweight, or overweight to obese. Cesarean deliveries in the U.S. also cost about $9500 more than vaginal births, on average, for an added $38 billion in delivery costs in the U.S. per year [22]. With 2.5% of all births in the U.S. delivered via Cesarean upon maternal request without any medical indication, nearly $1 billion in excess delivery costs can be attributed to mothers requesting medically unnecessary Cesarean deliveries [22, 23].

This study aims to establish causal evidence on the role of Cesarean delivery on maternal postpartum weight by relying on a natural experiment using fetal malpresentation – any fetal body part position other than vertex, one of the main indicators for Cesarean delivery in uncomplicated low-risk pregnancies. As we discuss in the methods section below, fetal malpresentation satisfies the natural experiment framework for modeling causal impact of Cesarean delivery, because 1) its natural occurrence is essentially random (not chosen by the mother or her clinician, and unrelated to maternal weight prior to pregnancy or gestational weight gain), and 2) it nearly uniformly involves Cesarean delivery. Therefore, a statistical association of fetal malpresentation at delivery with maternal weight during the postpartum period can be interpreted, with a high degree of plausibility, as the causal effect of Cesarean delivery on maternal weight.

Nearly 19% of all primary Cesarean deliveries are due to fetal malpresentation [2], which occurs in approximately 3% of all term deliveries, with breech presentation (bottom/feet down) being the most common form of fetal malpresentation [24]. Consistent with a large body of evidence documenting inferior health outcomes among malpresenting infants delivered vaginally relative to Cesarean [25–27] and because malpresentation is an indication for Cesarean delivery and thus recommended by the American Congress of Obstetricians and Gynecologists’ (ACOG), [28] most term malpresenting fetuses are delivered by Cesarean in the U.S.^1^

## Methods

### Study design

The study is a secondary retrospective data analysis of multi-hospital administrative data on first-time mothers with two births, using pre-pregnancy weight in the second pregnancy as the measure of maternal postpartum weight.

Our study contributes to the literature by relying on the conditional randomness of fetal malpresentation to isolate the causal impact of Cesarean delivery on maternal postpartum weight. As we point out above, in order for fetal malpresentation to serve as a natural experiment for measuring the effect of Cesarean delivery on postpartum weight, occurrence of fetal malplresentation must be uncorrelated with the mother’s weight prior to pregnancy and gestational weight gain, as well as with any other obesity risk factors and maternal characteristics that might have a direct influence on maternal post-pregnancy weight. Statistically, this condition is satisfied for approximately 85 to 91% of breech presentations where no cause or risk factor can be identified [29]. However, malpresentation is significantly more likely in earlier gestational age, and among shorter and older women, which are all factors known to be independently associated with maternal weight [29–32]. Some evidence also suggests potential additional risk factors for fetal malplresentation (altered intrauterine contour or volume, as well altered fetal shape or mobility), all of which could have unknown independent associations with post-partum weight [30, 33–38]. Although most of these confounding associations become statistically insignificant when gestational age at birth is included in the models [31, 37], use of fetal malpresentation as a natural experiment for Cesarean delivery requires adjusting for these potential confounders—so that, after the adjustment, malpresentation is randomly assigned.

To further address potential issues of confounding, we employ a propensity score matching method, matching first-time mothers who delivered by Cesarean where fetal malpresentation is the *only* clinical indicator to first-time mothers who delivered vaginally.

### Data and sample

#### Data

We relied on data previously obtained from PeriData.Net®, a data platform that contains data covering about 92% of hospital births in Wisconsin from 2006 to 2013. Following IRB approval at Marquette University (Weiss, PI) and data use agreements with 31 hospitals, we analyzed a de-identified dataset containing 236,820 birth records (64.78% of the total database) over the study period.

We restricted the sample to women for whom we observe *both* their first and second births with non-missing values on all key measures (see Appendix). We included mothers greater than age 17 at the time of the first birth and whose first birth was a singleton delivered between 28 and 42 weeks of gestation.^2^ We excluded women who delivered their first infant by Cesarean due to any clinical indication other than fetal malpresentation. Fetal malpresentation is listed as a clinical indication for Cesarean delivery only (it is not observable in the records for vaginal deliveries). Our analytic sample of first-time mothers with a second birth includes 29,463 women across 31 hospitals: 777 Cesarean deliveries due to fetal malpresentation (2.64% of sample) and 28,686 vaginal deliveries (97.36% of sample).

We examined the following maternal weight outcomes: the mother’s weight (in pounds), body mass index (BMI), and whether she was obese (BMI > 30) at the beginning of pregnancy 2; we refer to these measures as post-pregnancy or postpartum (to pregnancy 1). Pre-pregnancy weight for each pregnancy was derived from prenatal records submitted to the hospitals by the obstetrical provider prior to the birth. This measure was either self-reported to the obstetrical provider by the mother or measured at the first prenatal visit, but the method of obtaining the pre-pregnancy weight measure was not submitted. Maternal weight at delivery was recorded by hospital personnel as the weight measured either on admission in labor or at the last prenatal visit.

We included the following measures as matching characteristics in propensity score models: maternal age, maternal height, an indicator for any congenital anomalies of the newborn, perinatal death, or diagnosed fetal anomalies prior to delivery, an indicator for any uterine or placenta anomalies (any uterine or cervical anomaly, inverted uterus, incompetent cervix, placenta previa, polyhydramnios or oligohydramnios, eclampsia, uterine or cervical bleeding during the pregnancy), weeks of gestation at delivery, whether the mother reported alcohol or tobacco use during pregnancy, and whether the mother used the Women's, Infants, and Children (WIC) nutritional assistance program during the pregnancy, length of the interpregnancy interval (months between delivery of pregnancy 1 and pregnancy 2), and a set of maternal socio-demographic measures, including an indicator for advanced maternal age (> 35 years), indicators for white race, Hispanic/Latino ethnicity, college degree, and two sources of payment for the delivery (private health insurance, Medicaid, and other as the reference).

### Statistical analysis

We used a propensity score matching (PSM) model in order to restrict the sample to mothers with fetal malpresentation matched one-to-one to statistically similar mothers who delivered vaginally [39]. We did this by using a regression-adjusted nearest-neighbor method for matching without replacement. Specifically, we estimated the propensity score, obtained as the predicted values after estimating a logistic regression of fetal malpresentation on maternal pre-pregnancy weight, weight at delivery, malpresentation risk factors (age, height, weeks of gestation, uterine/cervical abnormalities, and fetal/congenital anomalies) and other health and socio-demographic measures described above. We then selected comparison mothers who had vaginal deliveries that are “nearest,” based on the estimated propensity score, to each treatment mother who delivered via Cesarean. We conducted t-tests to compare weight outcomes of treatment (Cesarean delivery due to fetal malpresentation) and comparison (vaginal delivery) mothers. Two-sided *p*-values of less than 0.05 were considered to indicate statistical significance.

We examined the robustness of our results by allowing multiple comparison mothers per treatment mother (instead of just one-to-one matching) and using propensity scores as weights in multivariate regressions. Additionally, as a final check, we excluded all mothers (both Cesarean due to fetal malpresentation and vaginal delivery) with any uterine, placental, or fetal/congenital anomalies, which reduced our sample by 7%.

## Results

### Sample characteristics

We present descriptive statistics of our analytic sample of first-time mothers with at least two births in the full unadjusted sample (first set of columns) and among the propensity score matched sample (second set of columns) in Table 1. The full sample included 29,463 women across 31 hospitals: 777 Cesarean deliveries due to fetal malpresentation (2.64% of sample) and 28,686 vaginal deliveries. This rate of Cesarean delivery due to fetal malpresentation is similar to the national rate among all hospital births in the U.S. that ended in a Cesarean section due to malpresentation in 2013.^3^

**Table 1:** Sample Characteristics, Comparison by Mode of Delivery for Unmatched (full) and Propensity Score Matched Samples

	Cesarean *n* = 777		Full Sample: Vaginal *n* = 28,686		*p*-value	Propensity Score Matched Sample: Vaginal *n* = 777		*p*-value
Maternal Weight Measures	Mean/%	SE	Mean/%	SE		Mean/%	SE	
Prior to Pregnancy 1								
Weight (pounds)	154.81	1.41	151.75	0.22	*0.02*	155.30	1.37	*0.80*
BMI	25.88	0.22	25.43	0.03	*0.03*	26.62	0.23	*0.80*
% Obese (BMI > = 30)	18.92	1.40	18.31	0.23	*0.66*	21.10	1.47	*0.28*
At Pregnancy 1 Delivery								
Weight (pounds)	187.83	1.37	183.65	0.22	*0.00*	187.85	1.33	*0.99*
*Malpresentation Risk Factors*								
Maternal age	28.79	0.17	26.68	0.03	*0.00*	28.88	0.17	*0.69*
% Maternal age > 35	9.40	1.05	4.92	0.13	*0.00*	9.27	1.04	*0.93*
Maternal height (inches)	64.82	0.11	64.76	0.02	*0.58*	64.86	0.1	*0.75*
Weeks of gestation at delivery	38.33	0.08	39.06	0.01	*0.00*	38.32	0.08	*0.9*
% Any fetal or congenital anomaly or infant death	6.43	0.88	2.86	0.09	*0.00*	6.94	0.91	*0.68*
% Any uterine or placental anomalies	13.51	0.01	4.33	0.12	*0.00*	13.90	1.24	
5-Minute APGAR	8.86	0.03	8.89	0.00	*0.14*	3.85	0.03	*0.92*
Interpregnancy Interval (months)	29.44	0.45	30.06	0.08	*0.24*	29.49	0.51	*0.95*
% Tobacco use during pregnancy	9.91	1.07	13.13	0.20	*0.01*	10.17	1.08	*0.87*
% Alcohol use during pregnancy	0.64	0.29	0.64	0.05	*0.99*	0.51	0.26	*0.74*
% Maternal education = College+	48.39	1.79	33.50	0.28	*0.00*	46.85	1.79	*0.54*
% Maternal race = white	89.57	1.10	7.59	0.26	*0.00*	89.06	1.12	*0.74*
% Mother Hispanic/Latino	6.82	0.91	11.82	0.19	*0.00*	6.82	0.91	*1.00*
%Mother married	79.15	1.46	49.89	0.29	*0.00*	80.44	1.42	*0.53*
% Received WIC during pregnancy	14.16	1.25	19.43	0.23	*0.00*	11.45	1.14	*0.11*
% Payer = Private health insurance	65.00	1.71	38.94	0.29	*0.00*	65.51	1.71	*0.83*
% Payer = Medicaid	16.47	1.33	29.06	0.27	*0.00*	15.06	1.28	*0.44*

Notes: Cesarean delivery refers to births with fetal malpresentation as the only clinical indicator. P-values reflect whether sample means/percentages are statistically different by mode of delivery

In the first two columns in Table 1, we compared sample means/percentages across the full sample of mothers. Mothers who delivered by Cesarean due to fetal malpresentation tended to weigh more prior to pregnancy and at delivery, were older, delivered earlier, and had higher rates of any fetal or congenital anomalies and uterine, cervical or placental abnormalities. These differences became statistically insignificant in the propensity score matched sample (last columns in Table 1).

### Post-pregnancy maternal weight

The average maternal weight at the beginning of pregnancy 2 was 159.24 lb (95% CI, 156.38 to 162.09) for mothers who delivered by Cesarean due to fetal malpresentation (Panel B of Table 2). This is not statistically different for the matched mothers who delivered vaginally (*p*-value = 0.85). The average BMI at the beginning of pregnancy 2 was 26.54 (95% CI, 26.08 to 26.99) and 26.62 (95% CI, 26.17 to 27.06) for mothers who delivered by Cesarean and vaginally, respectively; these are not statistically different (p-value = 0.80). There was also no significant difference in the percent of mothers who were classified as obese at the beginning of pregnancy 2 by mode of delivery: 22.91% (95% CI, 19.95 to 25.87) of mothers delivering by Cesarean relative to 24.20% (95% CI, 21.17 to 27.21) of mothers delivering vaginally (*p* = 0.55).

**Table 2:** Predicted Maternal Weight Measures Prior to Pregnancy 2

	Weight (pounds)	BMI	% Obese (BMI > 30)
A. Full Sample, *n* = 29,463			
Cesarean Delivery	157.30	26.32	22.53
	(0.42)	(0.06)	(0.67)
Vaginal Delivery	158.05	26.46	24.03
	(0.22)	(0.04)	(0.24)
* P*-value (statistically different)	0.07	0.02	0.04
B. Propensity Score Matched Sample, *n* = 1554			
Cesarean Delivery	159.24	26.54	22.91
	(1.46)	(0.23)	(1.51)
Vaginal Delivery	159.61	26.62	24.20
	(1.43)	(0.23)	(1.56)
* P*-value (statistically different)	0.85	0.80	0.55

Notes: Robust standard errors in parentheses. The p-value listed corresponds to the difference between breech Cesarean and vaginal deliveries in each model (column)

### Sensitivity analyses

In column 1 of Table 3, we present means and 95% CIs by mode of delivery where we have allowed multiple comparison mothers for each treatment mother in the propensity score matching (instead of just a one-to-one match). In column 2, we present adjusted means and 95% CIs from a multivariate regression model using propensity scores as weights and adjusting for the full set of health and socio-economic covariates listed above (and in Table 1). Finally, in column 3, we present means and 95% CIs by mode of delivery where we have excluded all mothers with one of the following risk factors for fetal malpresentation: any congenital anomalies of the newborn, perinatal death, or diagnosed fetal anomalies prior to delivery, any uterine or cervical anomaly, inverted uterus, incompetent cervix, placenta previa, polyhydramnios or oligohydramnios, eclampsia, uterine or cervical bleeding during the pregnancy. The resulting sample included 651 mothers who delivered via Cesarean due to fetal malpresentation and their one-to-one matches. Results are largely consistent with those presented in Table 2.

**Table 3:** Sensitivity Checks

	Multiple comparisons	Propensity scores as weights	Restricted Sample
*Panel A: Maternal weight (pounds)*	(1)	(2)	(3)
Cesarean Delivery (Malpresentation)	159.24	158.88	159.08
	(157.54, 158.46)	(157.13, 160.63)	(157.36, 163.40)
Vaginal Delivery	158.00	159.98	160.38
	(158.46, 162.09)	(159.60, 160.36)	(157.36, 163.40)
* P*-value (statistically different)	0.39	0.18	0.55
*Panel B: Maternal BMI*			
Cesarean Delivery (Malpresentation)	26.54	26.42	26.54
	(26.08, 26.99)	(26.15, 26.70)	(26.06, 27.03)
Vaginal Delivery	26.46	26.69	26.81
	(26.39, 26.54)	(26.61, 26.76)	(26.34, 27.29)
* P*-value (statistically different)	0.75	0.04	0.44
*Panel C: Mother Obese*			
Cesarean Delivery (Malpresentation)	22.91%	24.04%	22.27%
	(19.95, 25.87)	(21.82, 26.27)	(19.07, 25.48)
Vaginal Delivery	24.02	24.62%	25.19%
	(23.53, 24.51)	(24.11, 25.13)	(21.85, 28.54)
* P*-value (statistically different)	0.47	0.58	0.22
*N*	29,463	29,463	1302

Notes: Column 1 compares mean postpartum weight measures of mothers with fetal malpresentation Cesarean and vaginal delivery where we have allowed *multiple* comparison matches per treatment observation. Column 2 contains predicted means obtained after a multivariate regression model using the propensity scores as weights and adjusting for covariates listed in the text. Column 3 compares mean postpartum weight measures using a matched sample that excludes mothers with malpresentation risk factors. The p-values listed correspond to the difference between Cesarean (due to fetal malpresentation) and vaginal deliveries in each model (column). The three panels (A-C) correspond to each weight measure

## Discussion

Our findings do not support the notion derived from cross-sectional studies that Cesarean delivery leads to greater maternal weight retention after pregnancy. Using a large dataset from the state of Wisconsin that included hospital administrative records that contain vital records data, we found no evidence that first-time mothers delivering via Cesarean due *only* to fetal malpresentation weighed more at the beginning of their second pregnancies relative to their statistically matched mothers who delivered vaginally.

Our study contributes to both the literature examining the effects of Cesarean deliveries and to the literature endeavoring to explain the obesity epidemic in the U.S. we contribute to the ongoing debate about the risks and benefits of Cesarean delivery. In particular, our study suggests that although there are certainly health risks of Cesarean delivery, contribution to maternal weight retention does not seem to be one of them. Although obesity remains a significant public health concern, our findings do not suggest that mode of delivery contributes to weight gain. Instead, previously documented correlations of maternal weight retention and mode of delivery were likely driven by correlations between pre-pregnancy weight, gestational weight gain, and risk factors for Cesareans. Our finding adds to the knowledge base regarding the evolution of perinatal weight and its link to longer-term weight retention, but more research is needed to determine the extent to which other characteristics of the perinatal period matter in explaining long-term weight.

We point out the following limitation of our study. First, women who deliver a malpresenting infant via Cesarean may be a biased (higher-risk) subset of all women with a malpresenting fetus at delivery, potentially causing a bias toward finding an association between Cesarean delivery and poorer health outcomes. To the extent that poor health outcomes are also associated with being overweight or obese, this bias would likely only strengthen our conclusion that Cesarean delivery does not causally impact weight in the long term. Additionally, due to the clinical recommendation that malpresenting infants be delivered via Cesarean, this bias is likely small. In the 2013 national birth data, only 0.01% of all births to first-time adult mothers delivering between 28 and 42 weeks of gestation were malpresenting fetuses delivered vaginally.^4^ An additional limitation of the analysis is the use of linked birth data from a single state, which may limit the generalizability of our findings. Results may be different among mothers who deliver via Cesarean but do not go on to have additional children, mothers in other states or for mothers with interpregnancy intervals longer than those available in the dataset. Finally, we note that excluding women who have Cesareans due to other clinical indications was necessary for the quasi-experimental design, but limits the generalizability of our findings.

## Conclusions

In the light of a well-documented pattern of weight increase between pregnancies, our findings have important policy implications. Women and providers should be aware that women’s postpartum weight changes are not influenced by the mode of delivery. Recommendations for restriction of exercise in the first weeks after Cesarean do not appear to contribute to additional weight retention between pregnancies; attention to interconceptional weight management is equally important for vaginal and Cesarean mothers. Reversing the pattern of maternal weight gain, through counselling and surveillance about weight management during the childbearing years and between pregnancies, could contribute to a more stable weight trajectory for women over the life course.

## Appendix

**Table 4:** Sample Construction

Criteria	# of Observations
Starting sample	236,820 births
1.Keep only if MONTHS_SINCE_LAST_LIVE_BIRTH = 0 or is missing (no prior births) or BIRTH==1	97,171 births
2. Drop Cesarean births where fetal malpresentation is not indicator	75,021 births
3. Keep mothers age 18+ at first birth	69,875 births
4. Keep only births delivered between 28 and 42 weeks of gestation	67,480 births
5. Keep only singleton births	66,674 births
6.Keep mothers for whom we observe both first and second births	58,926 births
7. Final Analytic Sample (mothers with 2 births)	29,463 mothers

## Footnotes

1 Among first-time mothers with a term malpresenting fetus, 92% delivered via Cesarean in 2013 based on the authors’ calculations using the 2013 National Vital Statistics Systems births data.

2 Restricting the sample to mothers who deliver full-term (between 37 and 42 weeks of gestation) yields similar results to those presented here.

3 Based on authors’ calculations using the National Vital Statistics System births data for 2013, 3.34% of all births were delivered via Cesarean due to malpresentation.

4 Authors’ calculations using the 2013 NVSS births data. We note that a few states are missing presentation information for a small fraction of births with the exception of Virginia (missing about 50%) and Wyoming (missing on 100%).
